# Effect of Gold Nanoparticles on the TLR2-Mediated Inflammatory Responses Induced by *Leptospira* in TLR2-Overexpressed HEK293 Cells

**DOI:** 10.3390/nano10122522

**Published:** 2020-12-16

**Authors:** Kanidta Sooklert, Chawikan Boonwong, Pattama Ekpo, Rojrit Rojanathanes, Kanitha Patarakul, Chintana Chirathaworn, Sasin Thamakaison, Amornpun Sereemaspun

**Affiliations:** 1 Nanomedicine Research Unit, Department of Anatomy, Faculty of Medicine, Chulalongkorn University, Bangkok 10330, Thailand; kanidta.s1986@gmail.com (K.S.); emmy.s.tham@gmail.com (S.T.); 2 Research Division, Faculty of Medicine Siriraj Hospital, Mahidol University, Bangkok 10700, Thailand; chawikan.boo@mahidol.edu (C.B.); pattama.ekp@mahidol.ac.th (P.E.); 3 Department of Chemistry, Faculty of Science, Chulalongkorn University, Bangkok 10330, Thailand; rojrit@hotmail.com; 4 Department of Microbiology, Faculty of Medicine, Chulalongkorn University, Bangkok 10330, Thailand; kpatarakul@gmail.com (K.P.); chintana.ch@chula.ac.th (C.C.); 5 Chula Vaccine Research Center (Chula VRC), Center of Excellence in Vaccine Research and Development, Chulalongkorn University, Pathumwan, Bangkok 10330, Thailand

**Keywords:** gold nanoparticle, TLR2, leptospirosis, size-dependent

## Abstract

*Leptospira* infection can cause potential hazards to human health by stimulating inflammation, which is mediated mainly through the Toll-like receptor 2 (TLR2) pathway. Gold nanoparticles (AuNPs) are promising for medical applications, as they display both bioinert and noncytotoxic characteristics. AuNPs have been shown to have the ability to modify immune responses. To understand the in vitro immunomodulatory effect of AuNPs in a *Leptospira* infection model, the activation of TLR2 expression was examined in HEK-Blue-hTLR2 cells treated with *Leptospira* serovars and/or AuNPs (10 and 20 nm). The ability of AuNPs to modulate an inflammatory response induced by *Leptospira* was examined in terms of transcript expression level modulation of three proinflammatory cytokines (tumor necrosis factor-α, interleukin (IL)-1β and IL-6) using two-stage quantitative real-time reverse transcriptase PCR. The results revealed that the administration of 10 nm AuNPs could augment the *Leptospira*-induced TLR2 signaling response and upregulate the expression of all three cytokine gene transcripts, whereas the 20 nm AuNPs attenuated the TLR2 activation and expression of proinflammatory cytokines. This indicates that AuNPs can modulate inflammatory parameters in *Leptospira* infection and different-sized AuNPs had different immunomodulatory functions in this model.

## 1. Introduction

Leptospirosis is a zoonotic disease caused by infection of *Leptospira* bacteria. Depending on the *Leptospira* serovar and amount of bacterial infection, clinical manifestations can vary with the infected person. The symptoms of leptospirosis range from asymptomatic to severe symptoms that may even result in death [1]. The innate immune system responds to *Leptospira* infection through an inflammatory response as well as the production of cytokines. Uncontrolled production of cytokines can lead to a cytokine storm, causing immunoparalysis, which in turn causes sepsis and organ failure [2].

The innate immune system, also known as the nonspecific immune system or the first line of defense, is mediated through a limited number of germline-encoded pattern recognition receptors. Toll-like receptors (TLRs) are important members of the first line of defense against invasion of unknown materials. Currently, 13 TLRs have been identified in mammals and can be divided into the two groups of cell surface and intracellular organelle TLRs [3]. The activation of TLRs triggers downstream signaling, which results in stimulation of NF-kB signaling [4,5]. TLR pathway activation is also associated with the upregulation of genes encoding proinflammatory cytokines, thereby initiating inflammation [6,7]. A previous study revealed that the increased level of inflammatory cytokines and chemokines in response to *Leptospira* infection in humans was mediated mainly through the TLR2 pathway, rather than through the TLR4 pathway [8]. Thus, regulation of TLR2 responses in *Leptospira*-infected humans could potentially offer great promise for the control of inflammation.

While several compounds are known to stimulate TLRs, few have been observed to attenuate TLR signaling and none have been clinically approved as TLR inhibitors [9]. A possible new solution based on recent advances in nanotechnology is the use of nanodevices in controlling immune responses. Among various nanoparticles (NPs), gold NPs (AuNPs) have shown great promise in medical applications, and display both bioinert and noncytotoxic characteristics [10]. The use of AuNPs in medical products has become popular due not only to their biocompatibility but also to their diversity in size and function depending on the reaction conditions [11]. AuNPs are used in clinical diagnostic tests for different diseases, including leptospirosis, and are also used for their anti-inflammatory properties [12,13,14]. It is, however, unknown how AuNPs impact the immune response in leptospirosis.

In order to better understand the properties of AuNPs in the regulation of the TLR2-mediated production of proinflammatory cytokines, a comprehensive study on the effect of AuNPs on *Leptospira*-induced cytokines production through a TLR2-mediated pathway was performed in this study.

## 2. Materials and Methods

### 2.1. Characterization of AuNPs

AuNPs sizes 10 and 20 nm in diameter in an aqueous buffer containing sodium citrate as the stabilizer were purchased from Sigma-Aldrich (St. Louis, MO, USA). The particle size and shape of AuNPs were determined by transmission electron microscopy (TEM; Hitachi, Japan) and dynamic light scattering (DLS; Malvern Instruments, Malvern, UK). The particle surface charges and absorption wavelength of the commercial AuNPs solution were measured by the zeta potential Malvern Zetasizer Nano Series (Malvern Instruments, Malvern, UK) and ultraviolet (UV) spectrophotometer (Beckman Coulter, Brea, CA, USA), respectively.

### 2.2. Cell Culture

**Figure 1:**
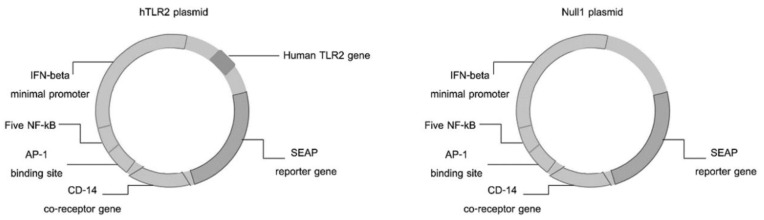
Diagram of the hTLR2 and Null1 plasmids for the cotransfection of the hTLR2 and SEAP reporter genes into HEK293 cells and SEAP without hTLR2 for the Null 1 cell line.

HEK-Blue-hTLR2 and HEK-Blue-Null1 cell lines (Catalog code. hkb-htlr2 and hkb-null1) were purchased from InvivoGen (San Diego, CA, USA). The HEK-Blue-hTLR2 cell line was designed to study the stimulation of human (h)TLR2 by monitoring the activation of NF-kB. These cells were obtained by cotransfection of the hTLR2 and SEAP (secreted embryonic alkaline phosphatase) reporter genes into HEK293 cells. The HEK-Blue-Null1 cells line also expresses the AP-1-inducible SEAP reporter gene under the control of the IFN-β minimal promoter fused to NF-kB, but do not encode for the hTLR2. The schematic diagram of the respective cotransfection plasmids for transformation of these cell lines is shown in Figure 1. HEK-Blue-hTLR2 and HEK-Blue-Null1 cells were cultured following the manufacturer’s instructions in complete medium (CM), comprised of Dulbecco’s modified Eagle’s medium (Sigma, USA) supplemented with 10% (*v*/*v*) heat-inactivated fetal bovine serum (Gibco, Waltham, MA, USA), 50 U/mL penicillin, 50 µg/mL streptomycin (Gibco, Waltham, MA, USA), 100 µg/mL Normocin (Invivogen, San Diego, CA, USA) and either 1X HEK-Blue™ Selection (Invivogen, San Diego, CA, USA) or 100 µg/mL of Zeocin (Invivogen, San Diego, CA, USA) for HEK-Blue-hTLR2 or HEK-Blue-Null1 cells, respectively. Cells were maintained at 37 °C in a humidified incubator with 5% (*v*/*v*) CO_2_ (Esco, Singapore).

### 2.3. Leptospira Culture Condition

The pathogenic *Leptospira interrogans* serovars Bratislava, Autumnalis and Pyrogenes and the nonpathogenic *Leptospira biflexa* serovar Patoc were obtained from the Department of Immunology, Faculty of Medicine, Chulalongkorn Hospital, Chulalongkorn University. Each *Leptospira* serovar was cultivated in Ellinghausen–McCullough–Johnson–Harris (EMJH) medium at 30 °C under aerobic conditions and grown until the cell density reached approximately 10^8^ cell/mL before harvesting by centrifugation.

### 2.4. Cell Viability Assay

HEK-Blue-hTLR2 and HEK-Blue-Null1 cells were seeded into 96-well plates at a density of 5 × 10^3^ cells/well in 45 µL of CM and incubated at 37 °C under 5% (*v*/*v*) CO_2_ for 12 h. The cells were treated with various concentrations of AuNPs (0 (control), 10, 30, 50, 80 and 100 ppm) and/or one of the respective pathogenic *L. interrogans* or nonpathogenic *L. biflexa* serovars at a multiplicity of infection (MOI) of 0 (control) or 100 (100 *Leptospira* per host cell) for 24 h. The cells were then incubated with 10 µL PrestoBlue™ reagent (Invitrogen, USA) for 30 min. The viability of the cells was then evaluated by measurement of the fluorescence intensity using a Varioskan Flash microplate reader at 560 and 590 nm (Thermo Scientific, Waltham, MA, USA), and expressed as the relative percentage cell viability to that of the control (100%). In addition, because it was possible for the nanoparticles to interfere with the spectrofluorometric assays, the same protocol was performed without cell seeding to confirm the accurate assessment of toxicity.

### 2.5. Cell Morphology

HEK-Blue-hTLR2 and HEK-Blue-Null1 cells were seeded into 24-well plates at a density of 5 × 10^4^ cells/well in 500 µL of CM. After incubation at 37 °C under 5% (*v*/*v*) CO_2_ atmosphere overnight, the cells were treated with noncytotoxic concentrations (derived from Section 2.5) of the respective sized AuNPs and/or *Leptospira* serovar and incubated for 24 h. The morphology of the cells was then examined under a phase contrast inverted microscope (Nikon, Eclipse TS 100, Tokyo, Japan).

### 2.6. Generation of Reactive Oxygen Species (ROS)

HEK-Blue-hTLR2 and HEK-Blue-Null1 cells were seeded into 96-black well plates at a density of 5 × 10^3^ cells/well in 100 µL of CM and incubated at 37 °C under 5% (*v*/*v*) CO_2_ for 12 h. The cells were then treated with 10 µM H_2_O_2_ as a positive control, 100 µM vitamin C (an antioxidant known to quench ROS), AuNPs of the respective sizes and/or the respective *Leptospira* serovar for 24 h. Cells were then washed with phosphate buffer saline (PBS) and incubated with 0.1 µM of 2′, 7′-dichlofluorescein-diacetate (H2DCF-DA; Molecular probes, Thermo Scientific, Waltham, MA, USA) at a volume of 100 µL/well for 30 min. After cells were washed with PBS and 200 µL of PBS was added to each well, the fluorescence of the dichlorofluorescein (DCF) product was measured, which is representative of intracellular ROS, using a microplate reader at an excitation and emission wavelength of 485 and 528 nm, respectively (Thermo Scientific, Waltham, MA, USA).

### 2.7. Activation of TLR: Detection and Quantification of Alkaline Phosphatase Levels

HEK-Blue-hTLR2 and HEK-Blue-Null1 cells were seeded into 96-well plates at a density of 5 × 10^4^ cells/well in 150 µL of CM and incubated at 37 °C under 5% (*v*/*v*) CO_2_ overnight before the addition of the respective sizes of AuNPs and/or one of the four *Leptospira* serovars and incubated at 37 °C in 5% (*v*/*v*) CO_2_ for 24 h. The levels of induced SEAP were determined using the QUANTI-Blue (Invivogen, San Diego, CA, USA) colorimetric enzyme assay. In brief, 20 µL of cell culture supernatant was collected and added to 180 µL of QUANTI-Blue^TM^, mixed and incubated at 37 °C in 5% (*v*/*v*) CO_2_ for 1 h. The mixtures change to a purple-blue color in the presence of SEAP, which was quantified by reading the OD at 620–650 nm using a microplate reader. Stimulation of the TLR-2 activates NF-kB and AP-1, which induces the production of SEAP. The HEK-Blue-Null1 cell line, which does not express hTLR2, was used as a negative control cell line. The level of SEAP was used to determine the amount of TLR2 activation, which also represents the levels of NF-kB activation.

### 2.8. RNA Extraction and Transcript Expression Level by Two-Stage Quantitative Real-Time Reverse Transcriptase (qrt-RT)-PCR

HEK-Blue-hTLR2 cells were seeded into 24-well plates at a density of 10^5^ cells/well in 500 µL of CM and incubated at 37 °C under a 5% (*v*/*v*) CO_2_ atmosphere overnight. The cells were treated with AuNPs of the respective sizes and/or one of the four *Leptospira* serovars and incubated for 24 h. The transcript levels of the respective genes were then evaluated by two-stage qrtRT-PCR. The total RNA was isolated from cells using TRIzol reagent (Invitrogen, Waltham, MA, USA) and in the first stage RT reaction, 1 µg of total RNA from each sample was collected for cDNA synthesis using a First Strand cDNA Synthesis Kit (Roche, Penzberg, Upper Bavaria, Germany) according to the manufacturer’s instructions. The second stage qrt-PCR was performed on the Express SYBR GreenER qPCR Supermix Universal (Invitrogen, Waltham, MA, USA) using the gene specific primers (Table 1) for TNF-α, IL-1β, IL-6, TLR2 and the housekeeping GADPH gene as an internal reference standard. The transcript expression level of each target gene was then expressed relative to that of the GADPH gene.

The enzyme was first activated by treatment at 50 °C for 2 min and 95 °C for 2 min and then thermal cycled at 95 °C for 10 min, followed by 40 cycles of 95 °C 15 s, annealing temperature (Table 1) for 1 min and 72 °C for 3 min using a StepOnePlus, Real-Time PCR System (ABI Applied Biosystems, Waltham, MA, USA). The CT (threshold cycle) values obtained for each respective target gene were normalized to the endogenous GAPDH level (housekeeping gene) and relative to the normalized calibrator.

### 2.9. Statistical Analysis

Assays were performed in triplicate and the results were expressed as the mean ± one standard deviation (SD). Statistical significance of differences in the means was analyzed using one-way ANOVA (analysis of variance), followed by Tukey’s multiple comparison tests, accepting significance at the *p* ≤ 0.05 level.

## 3. Results

### 3.1. Characterization of the AuNPs

**Figure 2:**
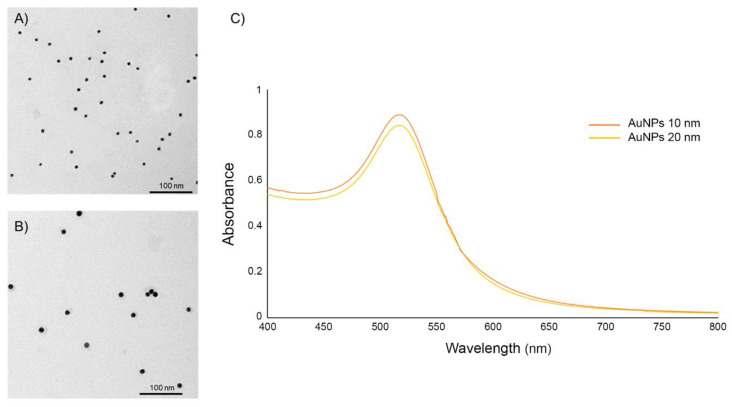
Representative images for the characterization of the (**A**) 10 nm and (**B**) 20 nm diameter AuNPs by TEM analyses of the monodisperse citrate-stabilized AuNPs and (**C**) UV-vis spectrometry.

The size and morphology of the AuNPs were observed by TEM analyses. TEM analysis revealed that the AuNPs had a spherical morphology, were well dispersed, and were approximately 10 nm (Figure 2A) and 20 nm (Figure 2B) in diameter. The results of the DLS analysis displayed that the average hydrodynamic diameter of the 10 nm and 20 nm commercial AuNPs solution in cell culture media supplemented with 10% FBS were 15.80 and 27.38 with polydispersity index (PdI) of 0.493 and 0.501, respectively (Table 2). The UV-Vis spectrum of 10 and 20 nm diameter AuNPs revealed a single wavelength peak between 500 and 550 nm (Figure 2C). Zeta potential measurements were found to be −37.3 and −31.3 mV for the 10 and 20 nm diameter particles, respectively (Table 2).

### 3.2. Cell Morphology and Viability Assay

**Figure 3:**
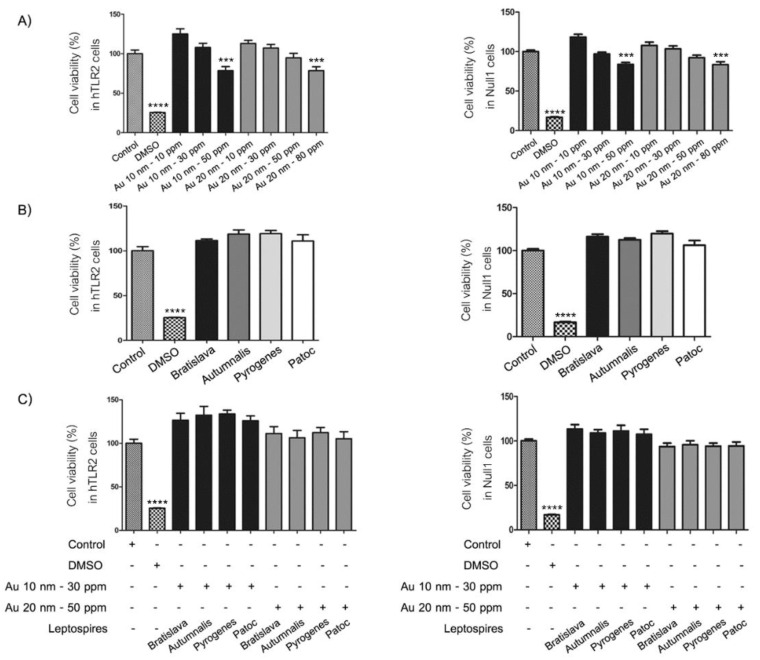
Effect of the (**A**) different size and concentrations of AuNPs, (**B**) different *Leptospira* serovars and (**C**) AuNPs in combination with different *Leptospira* serovars on the relative cell viability (% of control) of (left) HEK-Blue-hTLR2 and (right) HEK-Blue-Null1 cells after incubation for 24 h. Data are shown as the mean ± SD (*n* = 6). Significance of the difference compared to the control group is shown as *** and **** for *p* < 0.001 and *p* < 0.0001, respectively.

First, the noncytotoxic concentrations of the AuNPs and *Leptospira* serovars were evaluated to ascertain the appropriate or highest noncytotoxic concentrations for further use. To this end, for AuNPs, the cells were treated with AuNPs at concentrations of 0 (control), 10, 30 and 50 ppm for the 10 nm diameter AuNPs and 10, 20, 50 and 80 ppm for the 20 nm diameter AuNPs for 24 h. For the different *Leptospira* serovars, cells were treated at a MOI of 0 (control) or 100:1 for 24 h. In all cases, the relative (to the control) cell viability was then evaluated by the activity of mitochondrial dehydrogenase using the resazurin-based assay. In the plates without cells, the toxicity measured by PrestoBlue™ assay were not affected by exposure to AuNPs. In contrast, in the plates with cell culture, we found that exposure to increasing concentrations of AuNPs resulted in a numerical reduction in the relative cell viability in a dose-dependent manner, but this was only significantly less than the control at 50 and 80 ppm for the 10 and 20 nm AuNPs, respectively, (Figure 3A). Thus, the noncytotoxic concentration of 30 and 50 ppm for the 10 nm and 20 nm AuNPs, respectively, was used hereafter.

**Figure 4:**
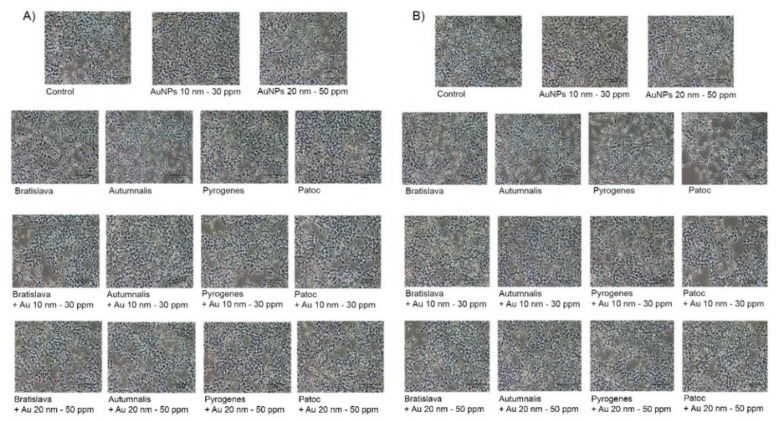
The morphological changes of AuNP-exposed HEK-Blue-hTLR2 cells (**A**) and HEK-Blue-Null1 cells (**B**) with and without *Leptospira* serovars under phase contrast microscopy.

For the *Leptospira* serovars, no obvious cytotoxicity against either the HEK-Blue-hTLR2 or HEK-Blue-Null1 cell lines were found for all serovars at a MOI of 100 (Figure 3B), while the percentage of viable cells was not significantly different between the control group and treated groups at the selected AuNP concentrations and serovars at a MOI of 100:1, although a numerically (but not significantly) slightly lower relative cell viability was noted with the 20 nm AuNPs than with the 10 nm AuNPs (Figure 3C). In addition, the evaluation of cytotoxicity by morphological study showed no difference in the cell morphology after any of the treatments compared to the control group in both cell lines (Figure 4). These data confirm that treatment with the various *Leptospira* serovars with the appropriate concentration of AuNPs did not adversely affect the cell viability and cell morphology of both cell lines.

### 3.3. Generation of ROS

**Figure 5:**
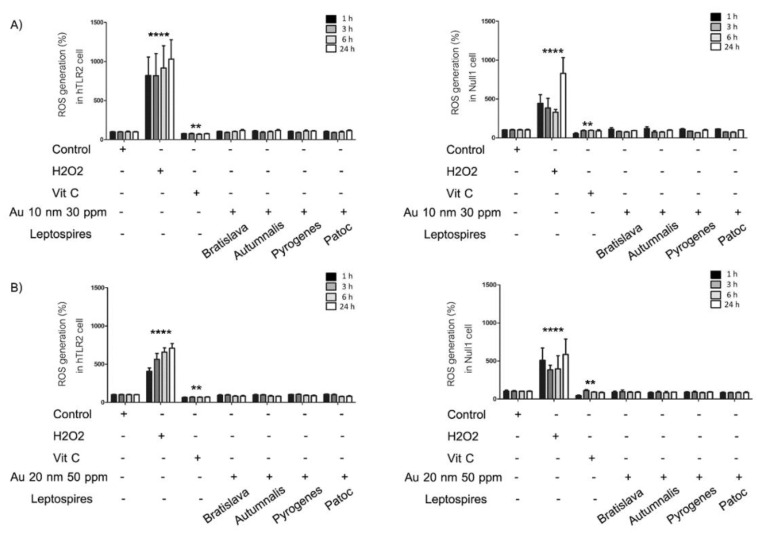
Effect of (**A**) 10 nm and (**B**) 20 nm AuNPs in combination with a different *Leptospira* serovar on the relative level (%) of reactive oxygen species (ROS) generation by (left) HEK-Blue-hTLR2 and (right) HEK-Blue-Null1 cells after 1, 3, 6 and 24 h. The level of ROS was detected using the DCFH-DA assay and expressing the fluorescence intensity relative to that for the control group. Data are shown as the mean ± SD (n = 6). Significance of the difference compared to the control group is shown as ** and **** for *p* < 0.01 and *p* < 0.0001, respectively.

With their inherently large surface area, nanomaterials display a higher reactivity compared to their bulk form. Exposure to certain nanomaterials may lead to an excessive production of reactive oxygen species (ROS), leading to a cascade of pathophysiological consequences, including inflammation induced by associated cell signaling pathways. To study the effect of AuNPs and/or *Leptospira* serovars on the generation of ROS following treatment in HEK-Blue-hTLR2 and HEK-Blue-Null1 cells for 1 to 24 h, the evaluation was performed using the DCFH-DA assay. Cells were treated with AuNPs (30 and 50 ppm for the 10 and 20 nm AuNPs, respectively) and the respective *Leptospira* serovars (MOI of 100:1) for 24 h, and then the relative level of ROS generation was evaluated. The level of ROS generation did not significantly differ between the control group and all treatment groups (Figure 5). In contrast, H_2_O_2_, as a positive control, significantly increased the ROS levels, while vitamin C, as an antioxidant and negative control, decreased the ROS generation.

### 3.4. Stimulation of hTLR2, Evaluated by Monitoring NF-kB Activation

HEK-Blue-hTLR2 cells were designed for studying the stimulation of hTLR2 by monitoring the activation of NF-kB. These cells were obtained by cotransfection of the hTLR2 and SEAP reporter genes into HEK293 cells. Stimulation with a TLR2 ligand activates NF-kB, which induces the production of SEAP. In contrast, HEK-Blue-Null1 (Null1) cells, the parental cell line of HEK-Blue-hTLR2 (Product descriptions, InvivoGen), express the SEAP reporter gene but not hTLR2.

**Figure 6:**
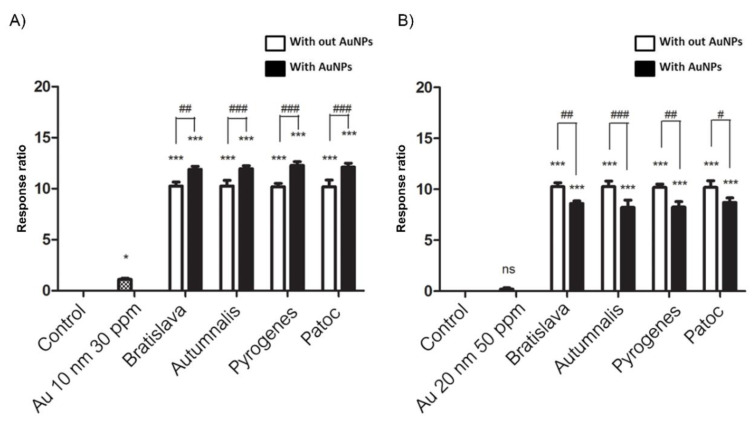
Effect of the (**A**) 10 nm (30 ppm) and (**B**) 20 nm (50 ppm) AuNPs on TLR activation with or without the presence of the different *Leptospira* serovars in HEK-Blue-hTLR2 cells. The levels of NF-kB-induced SEAP were determined by reading the OD at 655 nm and are relative (%) to that in the control group. Data are shown as the mean ± SD (*n* = 6). Significance of the difference compared to the control group is shown as * and *** for *p* < 0.05 and *p* < 0.001, respectively, and between with and without the presence of AuNPs as #, ## and ###, respectively.

Whether the AuNPs and/or *Leptospira* serovars activate NF-kB signaling through the TLR2 pathway was evaluated using the HEK-Blue-hTLR2 cells. The response ratios of NF-kB in HEK-Blue-hTLR2 were determined by subtraction and comparison with their negative controls. The result indicated that 10 nm AuNPs slightly, but significantly, stimulated SEAP secretion compared to the control, while each *Leptospira* serovar strongly induced SEAP secretion at approximately the same levels across each serovar. The combination of 10 nm AuNPs with each *Leptospira* serovar slightly, but significantly, further increased the SEAP secretion level compared to that with the respective *Leptospira* serovars only (Figure 6A). It must be noted that there was no significant difference in induced SEAP levels between the serovars, including between the pathogenic and nonpathogenic serovars.

In contrast, the 20 nm AuNPs alone did not induce SEAP secretion in the HEK-Blue- hTLR2 cell. Nonetheless, they slightly, but significantly, reduced the SEAP level induced by each *Leptospira* serovar (Figure 6B). As expected, the Null1 cells produced no SEAP secretion in all treatments, supporting that the observed SEAP secretion was mediated via activation of the hTLR2.

### 3.5. Proinflammatory mRNA Expression Levels

**Figure 7:**
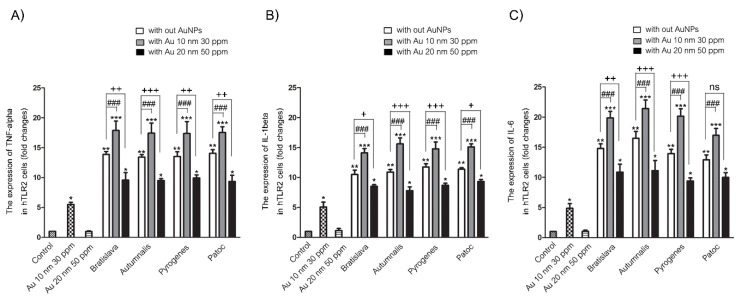
Effect of AuNPs and/or *Leptospira* serovars on the mRNA expression level of the proinflammatory cytokines (**A**) TNF-α, (**B**) IL-1β and (**C**) IL-6 in HEK-Blue-hTLR2 cells after 24 h treatment, as determined by two-stage qrt-RT-PCR and normalized to the GADPH housekeeping gene expression level. Data are shown as the mean ± SD (*n* = 6). Significance of the difference compared to the control group is shown as *, ** and *** for *p* < 0.05, *p* < 0.01 and *p* < 0.001, respectively. Significance of the difference between with and without the presence of 10 nm AuNPs is shown as ### for *p* < 0.001, respectively. Significance of the difference and between with and without the presence of 20 nm AuNPs is shown as +, ++ and +++ for *p* < 0.05, *p* < 0.01 and *p* < 0.001, respectively. ns = non-significant.

The transcript levels of the three evaluated proinflammatory cytokines (TNF-α, IL-1β and IL-6) were only minimally expressed in the unstimulated (control) HEK-Blue-hTLR2 cells, whereas they were all significantly upregulated after treatment for 24 h with 10 nm AuNPs and markedly and significantly upregulated by each of the different *Leptospira* serovars (Figure 7). The cotreatment with 10 nm AuNPs and *Leptospira* resulted in a significant increase in the cytokine transcript levels compared to that seen with each *Leptospira* serovar alone. However, the 20 nm AuNPs did not induce expression of any of these three gene transcripts, and in the copresence of each *Leptospira* serovar, the 20 nm AuNPs slightly, but significantly, reduced the transcript expression levels of all three cytokines compared to that induced by the respective *Leptospira* serovar alone. For each cytokine transcript, there was no significant difference in the induced expression level between the different *Leptospira* serovars. Thus, 10 nm AuNPs can induce TNF-α, IL-1β and IL-6 transcript expression and augment the response induced by *Leptospira* serovars, whereas 20 nm AuNPs cannot induce these cytokine transcript levels but significantly suppressed the response induced by the *Leptospira* serovars.

### 3.6. TLR2 mRNA Expression Level in Response to AuNPs

**Figure 8:**
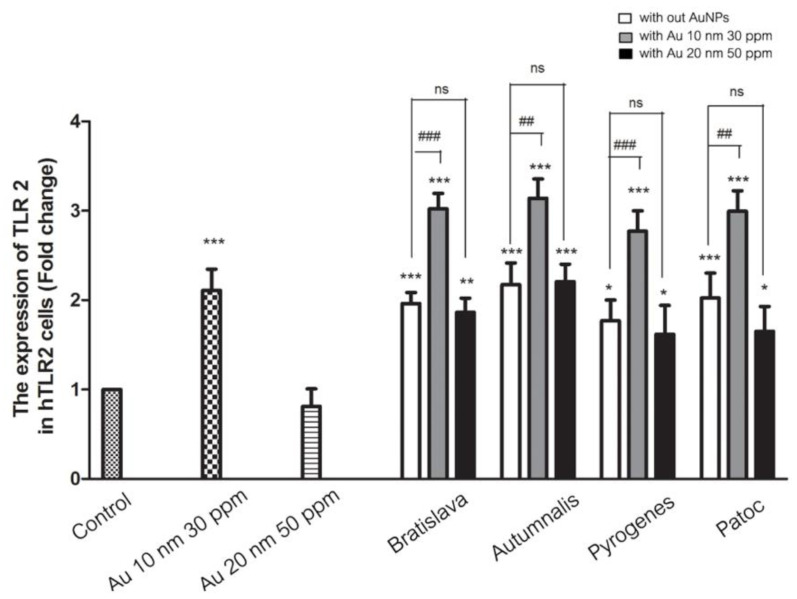
Effect of AuNPs on the *Leptospira*-induced TLR2 mRNA expression level in HEK-Blue-hTLR2 cells after 24 h treatment, as determined by two-stage qrt-RT-PCR and normalized to the GADPH housekeeping gene. Data are shown as the mean ± SD (*n* = 6). Significance of the difference compared to the control group is shown as *, ** and *** for *p* < 0.05, *p* < 0.01 and *p* < 0.001, respectively. Significance of the difference and between with and without the presence of 10 nm AuNPs is shown as ## and ### for *p* < 0.01 and *p* < 0.001, respectively. ns = non-significant.

Although 20 nm AuNPs can partially suppress the TNF-α, IL-1β and IL-6 transcript levels induced by *Leptospira*, it is not clear if that involved TLR2, which is upstream of the NF-kB pathway. Thus, the effect of AuNPs alone or with *Leptospira* on the TLR2 mRNA expression level in HEK-Blue-TLR2 cells was evaluated. The results showed that TLR2 mRNA was upregulated after treatment with 10 nm AuNPs, but not with 20 nm AuNPs. The hTLR2 transcript level was dramatically upregulated by each of the *Leptospira* serovars, and further upregulated in the copresence of the 10 nm AuNPs, but in contrast, it was partially suppressed by the copresence of the 20 nm AuNPs (Figure 8). Thus, the pattern of TLR2 mRNA expression in HEK-Blue-TLR2 cells after each treatment mirrored that of the TNF-α, IL-1β and IL-6 transcript levels.

## 4. Discussion

Over the past few decades, AuNPs have increasingly been used for their immunomodulatory effects, which includes their ability to control TLR activation levels [15,16]. The TLR2 is an important mediator of innate immune responses to *Leptospira* infections in human cells [2]. Binding of agonistic ligands, such as bacterial lipoproteins, to the TLR2 leads to the activation of NF-kB, which subsequently induces inflammatory gene expression [17]. In this study, we investigated the effect of AuNPs on the TLR2-mediated innate immune responses in the HEK-Blue-hTLR2 cell line. We found that the modulation of *Leptospira*-induced TLR2 signaling by AuNPs was dependent on the particle size, where the smaller AuNPs (10 nm) exhibited an immunoactivatory effect, whereas the larger AuNPs (20 nm) exerted an immunosuppressive effect. The TLR and proinflammatory cytokine activating effects exerted by the 10 nm AuNPs were not found with the 20 nm AuNPs, suggesting that 20 nm AuNPs are more biologically compatible than 10 nm AuNPs for application in modulating inflammatory response to *Leptospira* infection.

The mechanisms of *Leptospira*-induced immunoreaction have been reported in several studies, where activation of TLR2 is one of the major mediators involved in the inflammatory response [18,19,20]. Thus, we aimed to evaluate the effect of AuNPs on the *Leptospira*-induced TLR2-mediated innate immune responses using the commercially available HEK-Blue™/hTLR2 cell line. This cell is cotransfected with the hTLR2 and SEAP genes. Once the TLR2 ligand is stimulated, the activation of NF-κB and AP-1 induce the production of SEAP, and so the level of TLR2 activation can be easily detected using a SEAP detection assay.

No difference between the *Leptospira* serovars used in this study, which are pathogenic *Leptospira* (Bratislava, Autumnalis and Pyrogenes serovars) and nonpathogenic *Leptospira* (Patoc serovar), were found in the TLR2-mediated inflammatory response alone or in the copresence of AuNPs. This finding confirms previous knowledge that TLR2 is the main innate immune component which *Leptospira* uses as a mediator to induce inflammatory signals.

There have been many reports of nanomaterials, including AuNPs, that influence inflammatory signals [21,22,23,24,25]. However, few studies have focused on the effect of AuNPs on the TLR-mediated inflammatory responses. In this study, the immunological significance of AuNPs on the *Leptospira*-induced TLR2-mediated innate immunity was found, in which smaller AuNPs (10 nm) significantly increased TLR2 activation and proinflammatory cytokine expression levels, whereas larger AuNPs (20 nm) significantly reduced these phenomena. This finding is in accordance with previous studies that reported that smaller NPs have stronger effects on inducing activation of inflammatory responses than larger ones [26,27,28]. A previous study reported that 20 nm diameter AuNPs could reduce the expression of proinflammatory cytokines [29]. Additionally, small AuNPs (5 nm) were reported to significantly increase IL-1β and IL-6 mRNA expression levels in the mouse brain, while larger AuNPs (20 and 50 nm) did not [30]. These data suggest that the size of AuNPs is an important parameter in the inflammatory response modulation mechanism, which should be considered to make the application of AuNPs safer. In addition, our results revealed that the production of ROS, a key mediator of cellular inflammation, was not significantly changed in the *Leptospira* and/or AuNP treatments. These findings suggest that, depending on the particle size, AuNPs could act as an immunomodulator, independent of ROS generation levels.

The prominent physiochemical properties of NPs tend to be different from their bulk materials. The adjustment of these properties could provide either positive or negative advantages that could affect health upon exposure. There have been several reports on the size-dependent nature of NPs on TLR signaling and inflammatory responses, but the mechanism underlying the different sizes of AuNPs on inflammation is still controversial [31,32,33]. Although it is unclear how the different sizes of AuNPs interact with TLR2 in the presence of *Leptospira*, previous studies investigating the effect of AuNPs on TLR9 signaling in macrophages reported the size-dependent inhibition of TLR9 function, as smaller AuNPs are more effective in attenuating TLR9 signaling. In explaining this, a possible underlying mechanism may be that the large surface area of these smaller nanoparticles increases the probability of binding with their related proteins, which may result in the blocking of protein activities [34]. In addition, the use of 30 nm AuNPs in reducing LPS-induced eye inflammatory responses were reported. This may be attributed to the downregulation of TLR4 expression and its corresponding LPS-induced NF-κB activation [35].

The present study demonstrated the size-dependent immunomodulatory effect of AuNPs, with both pro- and anti-inflammatory effects depending on the particle size. However, the mechanism of action of AuNPs in the reduction and activation of TLR2 signaling is unknown. Moreover, the immune system response for AuNPs and leptospires is still unclear and should be assessed in greater detail. In this study, we use only citrate stabilizer and two sizes of AuNPs for evaluating the effect of AuNPs on inflammation in the *Leptospira*-infected model. To be improve the therapeutic efficacy of AuNPs for the treatment of inflammation and associated diseases, all of the physicochemical properties of AuNPs should be considered. Further studies should also be performed experiment to examine the protein produced by these proinflammatory mRNA genes after treatment.

## 5. Conclusions

In conclusion, this study confirmed that the immunomodulatory effect of AuNPs in the *Leptospira* infection model was dependent on the size of the AuNPs. Smaller AuNPs (10 nm) stimulated TLR2 signaling, resulting in an upregulated expression of the proinflammatory TNF-α, IL-1β and IL-6 gene transcripts. On the other hand, the larger AuNPs (20 nm) attenuated the TLR2 activation and partially downregulated the expression of these three proinflammatory gene transcripts. These results suggest the potential to design NP-based immunomodulators that can be applied to other bacterial infections that induce inflammation through TLR2 signaling. These findings indicate that AuNPs can be used as an immunotherapeutic agent in *Leptospira* infection and also raise the concern that same substances with different physicochemical properties could provide different biological reactions.

## Figures and Tables

**Table 1:** Primers used in the second stage qrt-PCR reaction.

Gene	Primer Sequence (5′ to 3′)	Annealing Temperature (°C)	Expected Amplicon Size (bp)
TNF-α	F: CCAGGCAGTCAGATCA	64	189
	R: TGGGAGTAGATGAGGTACAG		
IL-1β	F: TGGAGCAACAAGTGGTGT	57	157
	R: TTGGGATCTACACTCTCCAGC		
IL-6	F: TGCAGAAAAAGGCAAA	63	203
	R: CAACAACAATCTGAGGTG		
TLR-2	F: GGCCAGCAAATTACCTGTGTG	60	67
	R: AGGCGGACATCCTGAACCT		
GADPH	F: CAGGGGCCATCCACAGTCTTC	59	357
	R: CATCACCATCTTCCAGGAGCG		

**Table 2:** The zeta potential measurements of two different-sized AuNPs.

Nanoparticles (Dispersed in Culture Media Supplemented with 10% FBS)	pH	Zeta Potential (mV)	Hydrodynamic Diameter (nm)	PdI
10 nm AuNPs	7.28	−37.3 mV	15.80	0.493
20 nm AuNPs	7.31	−31.3 mV	27.38	0.501
